# RNA Binding Protein LIN28B: a prime time player shaping neuroblastoma aggression and metastasis

**DOI:** 10.18632/oncoscience.512

**Published:** 2020-06-08

**Authors:** Dongdong Chen, Robert W. Schnepp

**Affiliations:** ^1^Aflac Cancer and Blood Disorders Center, Department of Pediatrics, Emory University School of Medicine, Children’s Healthcare of Atlanta, Atlanta, GA, USA; ^2^Winship Cancer Institute, Emory University School of Medicine, Atlanta, GA, USA

**Keywords:** LIN28B, let-7, PBK, neuroblastoma, metastasis, RNA binding protein

Neuroblastoma, a pediatric cancer of the developing neural crest, is a strikingly heterogeneous disease, with clinical presentations ranging from the child with an asymptomatic abdominal mass to the patient presenting *in extremis* due to respiratory compromise [1]. At diagnosis, patients with high-risk disease generally have a primary (often abdominal) mass, as well as metastases in various sites, including the liver, bone marrow, cortical bone, and lymph nodes. Conceptually, most studies have focused on defining the biology and deregulated oncogenic signaling that promote growth of the primary tumor. In comparison, the mechanistic and molecular underpinnings of metastatic disease are less thoroughly explored.

Seeking to define novel drivers of neuroblastoma aggression, we utilized genome-wide association studies to identify an association between neuroblastoma susceptibility and germline variation in Lin-28 Homolog B (LIN28B) [2]. LIN28B is an RNA binding protein and one of its major functions is the inhibition of the maturation of the *let*-7 family of microRNAs [3, 4]. In addition, LIN28B binds directly to multiple RNA species, including mRNAs and non-coding RNAs [5, 6, 7]. In the physiological context, LIN28B promotes self-renewal, with let-7 opposing its effects [3]. In the oncogenic context, LIN28B is overexpressed in multiple adult and pediatric malignancies, including leukemias [8] ,colon [7, 9] and pancreatic cancer [10], neuroblastoma [2, 11-13], and Wilms tumor [14]. Moreover, tissue-specific overexpression of LIN28B in mouse models leads to the development of various cancers, cementing its role as an oncogenic driver.

In neuroblastoma, we initially showed that high *LIN28B* expression is correlated with higher stage neuroblastoma and suboptimal outcome [2]. Subsequently, we demonstrated that LIN28B promotes the proliferation of neuroblastoma cells and defined RAN GTPase and Aurora kinase A (AURKA) as novel genes regulated by LIN28 [12]. As LIN28B has been shown to promote metastasis in two adult epithelial malignancies, esophageal [15] and colon cancer [9], we speculated that it might also influence neuroblastoma metastasis. In a murine xenograft model of neuroblastoma metastasis, we demonstrated that LIN28B indeed promotes metastasis [16]. Further, we showed that it drives metastasis by increasing both self-renewal and migration; in contrast, let-7 opposes these processes.

**Figure 1:**
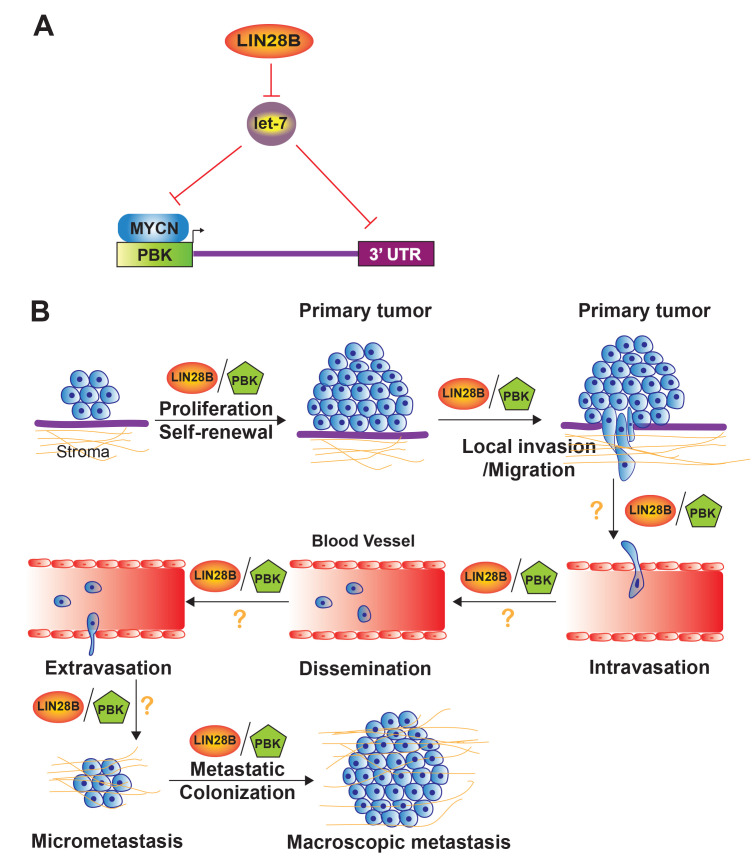
Model depicting the influence of LIN28B-PBK signaling on neuroblastoma metastasis. (A) A schematic diagram illustrates that two neuroblastoma oncogenes, LIN28B and MYCN, directly regulate the expression of PBK. LIN28B inhibits the maturation of the let-7 family of microRNAs, while let-7 binds the 3’ UTR of PBK and inhibits the expression of PBK. MYCN directly binds to the PBK promoter, increasing transcription. (B) A schematic shows that LIN28B/PBK signaling promotes proliferation, self-renewal, and local invasion/migration. We speculate that LIN28B and PBK may be involved in additional steps of metastasis, as illustrated. Illustration is inspired by schematics depicted in Valastyan *et al*. [25] and Saxena *et al*. [26]

Next, we sought to define downstream targets of LIN28B, focusing our efforts on novel genes that are therapeutically tractable. We found the expression of LIN28B and PDZ binding kinase (PBK) expression to be strongly and positively correlated in tumors and focused our efforts on characterizing this interaction. PBK, also known as T-LAK cell-originated protein kinase (TOPK), is a serine/threonine kinase that promotes the self-renewal of neural stem cells; it is overexpressed in diverse adult histotypes and implicated in multiple hallmarks of cancer, including cell cycle regulation, apoptosis, and metastasis [17]. Our studies provided evidence that LIN28B promotes PBK expression and that PBK is both a LIN28B and a let-7 target (Figure 1A). We went on to additionally define PBK as a direct transcriptional target of MYCN, thus linking PBK to two neuroblastoma oncogenes, LIN28B and MYCN (Figure 1A). Finally, our studies illustrated that PBK promotes cell proliferation, self-renewal, and migration, phenocopying LIN28B (Figure 1B).

These studies then reveal a novel role for LIN28B and PBK in shaping neuroblastoma metastasis. At the same time, they suggest a number of directions for future investigations into LIN28B-PBK signaling. While our findings show that PBK lies downstream of both LIN28B and MYCN, and the functions of PBK mimic those of LIN28B *in vitro*, whether PBK sculpts neuroblastoma metastasis in the *in vivo* setting is unknown. Given the overlap between LIN28B and PBK in mediating self-renewal and migration, we hypothesize that both LIN28B and PBK promote neuroblastoma metastasis (Figure 1B). While our data support a role for LIN28B and PBK in cell proliferation, self-renewal, and migration, there are many additional steps in metastasis, including intravasation, dissemination, and extravasation, and it is reasonable to speculate that LIN28B and PBK may support these functions. While LIN28B remains a challenging target, PBK is currently therapeutically tractable and clinically relevant inhibitors, primarily targeting the kinase activity of PBK, exist. In preclinical models of colon [18] and ovarian cancer [19], PBK inhibition leads to decreased metastatic dissemination. Might targeting PBK pharmacologically, either as single-agent therapy or in combination with chemotherapy or other targeted agents, reduce metastatic burden?

Our current study, focused on LIN28B-PBK signaling, in conjunction with our previous discovery of a LIN28B-RAN-AURKA network, [12] highlights two kinases that are downstream LIN28B/let-7 targets in neuroblastoma. Interestingly, other investigators have identified a LIN28B-RAN-AURKA signaling axis in hepatoblastoma [20] and have defined Aurora kinase B (AURKB) as a molecular target of LIN28b/let-7 signaling [21]. Additionally, both LIN28A and B have been shown to regulate various components of the insulin-PI3K-mTOR pathway, promoting glucose metabolism [22]. These various studies demonstrate that LIN28 influences multiple kinases and suggests the possibility that it may exert activity across the kinome. High-throughput profiling of the kinome will help identify the full repertoire of LIN28B-influenced kinases, furthering our understanding of how LIN28B influences this pivotal class of proteins. Given the therapeutic tractability of kinases, such approaches may nominate potential novel therapeutic vulnerabilities. With the involvement of LIN28B in metastatic dissemination, might subsets of these kinases help drive metastatic spread and could inhibiting such kinases diminish dissemination?

With respect to the molecular mechanisms by which LIN28B influences the oncogenic phenotype, it is clear that its influence on let-7 processing is pivotal. However, LIN28B has also been shown to bind to mRNAs and other RNA species directly. There are comprehensive studies that have utilized techniques such as ribonucleoprotein cross-linking, immunoprecipitation, and high-throughput sequencing (CLIP-Seq) to define direct LIN28B targets. However, many of these studies were performed in cell lines chosen for their robustness in cell culture as opposed to their faithful modeling of various tumor histotypes [5, 6]. While these experiments have certainly yielded valuable insights, it will be of significant interest to utilize high-throughput technologies to identify LIN28B-bound mRNAs in the context of neuroblastoma and other histotypes. Ideally, such experiments would pair small RNA-Seq/RNA-Seq, CLIP-Seq/related technologies, and high-throughput proteomics approaches, allowing for the possibility of simultaneously studying the effects of LIN28B perturbation on the expression of mRNAs, RNA species, and protein.

Beyond the effects of LIN28B and let-7 on molecular signaling, there are significant discoveries to be made regarding the impact of LIN28B/let-7 on the metabolome, particularly in neuroblastoma and the other pediatric histotypes in which LIN28B has been implicated as an oncogenic driver. While elegant murine models have demonstrated the role of LIN28B/let-7 on fostering optimal glucose metabolism in the physiologic setting, our knowledge of how LIN28B/let-7 shapes metabolism in the oncogenic setting remains incomplete[22]. Future metabolomic profiling experiments in the context of neuroblastoma and other malignancies will help augment our knowledge of the roles of LIN28B in shaping the malignant phenotype. Moreover, metastasizing cells must adjust their metabolisms to differing environments, ranging from escape from the primary tumor, to migration through the bloodstream and other tissues, to establishing outgrowth in new environments [23]. In this regard, experiments profiling the effects of LIN28B on the metabolic phenotype of neuroblastoma cells in differing phases of the metastatic process (i.e. growing as tumorspheres, during invasion/migration, initial outgrowth in metastatic lesion), would likely provide insights into not only LIN28 biology, but also further our understanding of neuroblastoma metastasis itself.

**Figure 2:**
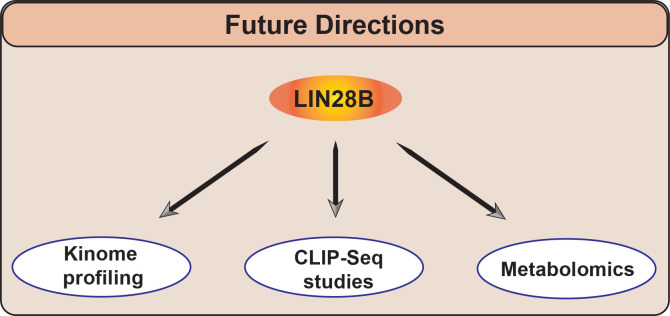
Future research directions to comprehensively define LIN28B functions. We propose high-throughput approaches, including kinome profiling and CLIP-Seq, to comprehensively identify LIN28B-influenced kinases and LIN28B- bound RNAs. Given the role of LIN28B in supporting glucose metabolism and the importance of metabolism in metastasis, metabolic profiling may help further our understanding of LIN28B function.

We have begun to develop a better understanding of how LIN28B shapes multiple hallmarks of the malignant phenotype, including cell proliferation, self-renewal, and metastasis. Additionally, we have outlined topics for further consideration, including how PBK affects metastasis, how LIN28B regulates the kinome, which targets are directly bound by LIN28B, and how LIN28B influences the metabolome (schematized in Figure 2). Taken together, such studies aim to clarify various cellular mechanisms by which LIN28B shapes neuroblastoma aggression. It is worth noting that, beyond LIN28B, there are approximately 1550 RNA binding proteins (RBPs) encoded in the human genome [24]. In addition to the research questions we have sketched out above, the potential contribution of these proteins to aggression in neuroblastoma and other tumors, is unknown, yet clearly of interest. We predict that such studies will nominate more “prime time” RBPs, providing a strong foundation for future efforts to drug this understudied class of proteins.
